# Experience of Health System Personnel in the Implementation of Mass Distribution Campaigns for the Control of Lymphatic Filariasis in Rural Guinea in 2022

**DOI:** 10.3390/tropicalmed9110265

**Published:** 2024-11-05

**Authors:** Akoi Zoumanigui, Delphin Kolié, Lamine Lamah, Nouhou Konkouré Diallo, Aissata Tounkara, Hawa Manet, Mamadou Camara, Alexandre Delamou

**Affiliations:** 1 Programme National de Lutte Contre les Maladies Tropicales Négligées à Chimiothérapie Préventive, Conakry P.O. Box 585, Guinea; dnoufr@yahoo.fr; 2 National Training and Research Centre in Rural Health, Maferinyah P.O. Box 2649, Guinea; dkolie@maferinyah.org (D.K.); hawa@maferinyah.org (H.M.); adelamou@maferinyah.org (A.D.); 3 Helen Keller International, Conakry P.O. Box 6050, Guinea; llamah@hki.org; 4 Centre of Excellence in the Prevention and Control of Communicable Diseases, University of Conakry, Conakry P.O. Box 1017, Guinea; atounkara@cea-pcmt.org; 5 Programme National de Lutte Contre les Maladies Tropicales Négligées à Prise en Charge des Cas, Conakry P.O. Box 585, Guinea; mamadycamarafr@yahoo.fr

**Keywords:** experience, mass distribution, lymphatic filariasis, neglected tropical diseases, guinea

## Abstract

This study documents the experiences of health system personnel in the implementation of mass distribution campaigns for the control of lymphatic filariasis in rural Guinea. This was an exploratory qualitative study using data collected from implementing actors of mass distribution campaigns in the Boké health district. The results showed four main facilitators of mass distribution campaign rollout in the Boké health district: (i) support to the district teams in the organization of the campaigns; (ii) involvement of community-based associations in social mobilization; (iii) strong adherence of the communities to the different mass distribution campaigns, facilitated through the involvement of community relays, who are members of these communities, in the distribution of drugs; and (iv) transparency in the allocation of incentives to drug dispensers and supervisors. However, the frequent shortages of medicines, the difficulty of access to rural areas, and the lack of logistical means for the supervision of activities were the main obstacles to the success of the various mass distribution campaigns in Boké. The provision of buffer stocks for special areas such as Boké by national programme actors and partners, joint planning of campaign activities with local managers of health systems and services, and improvement of existing mechanisms for motivating health workers, including community health workers, during future campaigns should help to achieve national objectives in the fight against NTDs in Guinea.

## 1. Introduction

Lymphatic filariasis is a parasitic disease transmitted to humans through the bite of an insect, the mosquito. The disease affects the lymphatic system, often resulting in abnormal enlargement of certain parts of the body such as the feet and scrotum. This is a severe disability and often causes social stigma [1]. According to recent estimates by the World Health Organization (WHO), about 17% of the world’s population, mainly in Africa and Southeast Asia, is at risk of lymphatic filariasis [2].

In recent years, enormous progress has been made in the control of filariasis worldwide, thanks in particular to repeated chemoprevention in countries endemic to the disease [1]. In 2018 alone, 51 million new filariasis infections were reported worldwide; a 74% decline in the incidence of cases since the global elimination programme began in 2000 [1].

The experience of lymphatic filariasis elimination is well documented in several Asian and North African countries [3,4,5]. Authors in Asia and Africa reported that the success of lymphatic filariasis elimination is linked to good collaboration between national and international actors as well as the integration of efforts to control all other neglected tropical diseases [3,4,5]. Other authors have also indicated that decentralization of the disease control activities at the community level and capacity building of local health facilities in the management of complicated forms (lymphedema, hydrocele, adenolymphangitis, etc.), including training and the provision of equipment and supervision of health personnel, have been important facilitators in the elimination of lymphatic filariasis in African and Western Pacific regions [4,5].

In Guinea, lymphatic filariasis was endemic to 24 out of 38 health districts in the country in 2013 [6]. Among these health districts, four were considered hyper-endemic, namely Boké (13%), Forécariah (11%), Kérouané (12%), and Beyla (14%) [6]. However, efforts deployed by the government have resulted in a reduction in disease prevalence in most endemic health districts, especially in Boké [7,8]. In fact, the disease’s prevalence dropped from 13% in 2013 to 0.32% in 2021 in the Boké health district, according to the results of a preliminary assessment survey of lymphatic filariasis transmission [7]. Similarly, another assessment survey conducted in 2022 found no cases of lymphatic filariasis among nearly 1600 children sampled and tested in the Boké health district [8]. Despite this success, little is known about the determinants of the success of lymphatic filariasis control in the Boké health district. This study was therefore initiated to document the experience of health system personnel in Boké in implementing mass distribution campaigns for lymphatic filariasis control. Specifically, this study aimed to explore health system personnel’s perceptions about the rollout of mass distribution campaigns and describe best practices and obstacles that health system personnel face in implementing these mass distribution campaigns. The findings of this study could provide relevant information for the control and elimination of lymphatic filariasis in the country as well as other NTDs in Guinea and countries with similar contexts.

## 2. Materials and Methods

### 2.1. Type and Period of Study

This study was an explanatory qualitative study to document the experience of implementing health system personnel in mass distribution campaigns against lymphatic filariasis in the Boké health district, in Guinea, in 2022.

### 2.2. Study Settings

#### 2.2.1. General Parameters

The Republic of Guinea is located in West Africa and has 38 health districts. Its population was estimated at 13 million in 2022 [9,10]. Most of the population live in rural areas (65%) and beneath the poverty line (55%). The health system is tiered to a primary level (413 health centers and 726 health posts), a secondary level (26 district hospitals, 7 regional hospitals, and 8 medico-communal centers), and a tertiary level (3 national reference hospitals) of health facilities [11].

#### 2.2.2. Specific Parameters

The study was conducted in the health district of Boké. This rural health district is located nearly 250 kilometers from the capital city, Conakry. It had an estimated population of 568 thousand inhabitants in 2022 [9]. Agriculture, fishing, and animal breeding are the main activities carried out by the population of the Boké health district.

**Figure 1:**
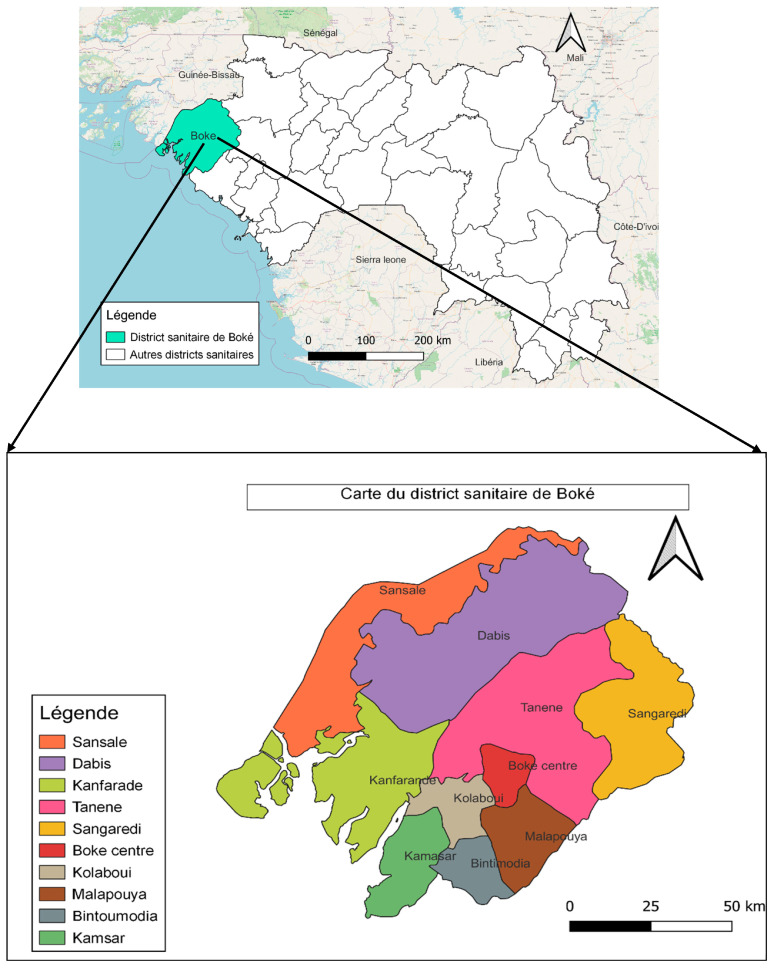
Study sites.

The local health system includes a health district management team, a district hospital, 13 health centers (10 of which are in rural areas), and 53 health posts (see Figure 1—Map of Boké and the study sites).

#### 2.2.3. Lymphatic Filariasis Control in Guinea

The strategy for lymphatic filariasis control in Guinea is in line with WHO recommendations and consists of preventive (chemotherapy) and curative (management of cases) services. Significant efforts have been made in recent years to control lymphatic filariasis in Guinea. Among these efforts is the mass administration, on an annual basis, of Ivermectin and Albendazole to people aged 5 years and older in all endemic districts. During the five years of mass treatment against lymphatic filariasis, the health district of Boké has recorded very good epidemiological coverage of 65% or more [9]. With respect to the management of complications of lymphatic filariasis (lymphedema and hydroceles), training of community health workers was provided in 24 health districts for the detection of complications and the reporting of information at the 111 district and regional levels [10]. This was accompanied by the training of surgeons and 112 surgical staff in all regions endemic for the disease. The management of complications of lymphatic filariasis is individual at the level of the surgical services and it is the responsibility of patients since 2021 to present themselves for treatment.

### 2.3. Study Participants and Recruitment

According to the levels of the health pyramid in Guinea, three groups of participants were interviewed in this study: managers of the national programme for the control of NTDs at national, regional, and district levels; health service managers and healthcare providers; and social mobilizers involved in the distribution campaigns in Boké.

### 2.4. Data Collection Technique and Tool

Data were collected through in-depth individual interviews with the implementing actors (health personnel involved in the distribution of the drugs, Ivermectin and Albendazole). This interview guide was divided into four main sections following the research objectives (see Annex). The key informant interviews allowed us to better understand the best practices, facilitators, and challenges of implementing mass distribution campaigns for lymphatic filariasis control in the Boké health district. These key informants were selected based on their involvement in the implementation process of the lymphatic filariasis mass distribution campaigns in Boké. Interview guides were developed for the different stakeholder groups targeted in the study. The guides were developed based on the research questions of the study, including best practices adopted in the implementation of the campaigns, facilitators, and obstacles to the implementation of filariasis control campaigns in Boké. The interviews were recorded using a smartphone after obtaining permission from the participants.

### 2.5. Data Analysis

The interviews were transcribed verbatim and this was followed by thematic analysis. All the transcripts were coded manually using an Excel spreadsheet. A thematic analysis was applied using deductive and inductive approaches. The diversity of data sources contributed to the internal validity of the study through triangulation [12].

### 2.6. Ethical Considerations

The research protocol for this study was approved by the National Health Research Ethics Committee of Guinea (number: N-114-CNERS-22) before data collection began.

## 3. Results

### 3.1. Profile of Health System Personnel Interviewed in the Study

A total of 18 participants were interviewed for this study, including health system personnel from the regional and district management teams, skilled healthcare providers, and community health workers. Of the 18 participants interviewed, 14 (78%) were men and 4 were women (22%). Their characteristics are shown in Table 1.

### 3.2. Health System Personnel’s Perceptions of Facilitators for the Implementation of Mass Distribution Campaigns for Lymphatic Filariasis Control

Analysis of interviews with the study participants revealed four main facilitators of the implementation of mass distribution campaigns for lymphatic filariasis control in the Boké health district. The first reported facilitator was the support given to the district teams in organizing the campaigns. This support consisted of the mobilization of material, financial, and human resources necessary to conduct these campaigns. The availability of material, human, and financial resources during the mass distribution campaigns in the Boké health district enabled the teams to comply with the national directives required for the implementation of these campaigns, in particular the organization of cascade training involving, from the top to the bottom level, the national supervisors, the regional and district level trainers, the managers of the primary health care facilities (health centers), the proximity supervisors, and the community distributors. Another aspect of this support, according to the respondents, was the provision and distribution of work tools such as registers, height measurement tools, and medicines to community distributors.

The second facilitator of the successful implementation of mass distribution campaigns was the involvement of community-based associations such as women and youth groups as well as community leaders in social mobilization, as illustrated by the respondent below.

“…Sometimes, we involve women leaders and religious leaders in community mobilization… Each of these actors plays a role in removing community reluctance towards the campaigns… For example, if community health workers fail to remove the reluctance, they call on the sector chief, and so on up to the district supervisor…”

EIA 03, community health worker, male.

The third reported facilitator was the strong support of the communities for the various mass distribution campaigns. According to the respondents, medication adherence was facilitated by the involvement of the community health workers (who are members of these communities) in drug distribution. Respondents also mentioned that providing CHWs with picture boards facilitated their work of informing communities about the risks of lymphatic filariasis and their consequences.

“In 2019, in Fria and Coyah health districts people who received Praziquantel during the distribution campaign developed reactions [side effects]… If it had not been for the involvement of the communities [community health workers] in our various distribution campaigns, we would not have been able to overcome the reluctance [and get the communities to adhere].”

EIA 01, regional health system manager, male.

Finally, the fourth reported facilitator was the transparency involved in the allocation of field bonuses for drug distribution agents and supervisors. According to the respondents, this transparency in the allocation of field bonuses was key in motivating local health system actors to carry out the activities planned for the achievement of the campaign coverage objectives.

### 3.3. Best Practices Undertaken by Health System Personnel in Implementing Mass Distribution Campaigns Against Lymphatic Filariasis

Several best practices emerged from the interviews with respondents. First, it was emphasized that simulation sessions were organized for the distribution agents in the Boké health district. These simulation sessions included filling out the register for counting and distributing medicines, the attitude to adopt towards communities, including what clothing to wear, and the key messages to convey during community awareness-raising.

“…During the training, we break up the groups to do the simulation… We make sure that the distribution agents understand how to proceed in the field, through simulation sessions…”

EIA 03, community health worker, Male

“In carrying out the practice, [we have] charts on which the agents are trained on reporting the quantities of tablets used, how to proceed with tracing the registers and measure the height… We also do demonstrations on how to take the height, how to administer the tablets…”.

EIA 13, health center manager, female.

Second, another best practice identified was the adaptation of national planning and strategies to the local realities. For example, it was noted that health system personnel in the Boké health district recruited additional community supervisors to cover all endemic localities, including those omitted in the initial planning by the central-level actors. In such circumstances, the initial planned field bonuses were distributed to all supervisors, including those mobilized at the last minute.

“…Sometimes also, some remote areas are omitted from the national [NTD] program planning, due to lack of resources. In such circumstances, we rework the composition of the teams and redeploy supervisors and distribution agents… We also ensure the provision of vehicles and fuel to these teams to cover the omitted areas… If two supervisors were originally planned, but we have a need for five supervisors, we deploy the five supervisors and redistribute the field bonuses of the two supervisors to the five who actually participated in the work.”

EIA 01, regional health system manager, male.

It was also reported that some health center managers used their facility’s revenues to support the deployment and motivation of distribution agents in hard-to-reach locations. For example, the participants mentioned that due to the limited number of inhabitants in some villages, central or district team planning would rarely take these locations into account. In these contexts, health center managers would deploy additional teams of distribution agents from their revenue to cover these locations and allow for coverage of their entire health area.

“When a health centre chief reports such a situation to us; we tell him to draw on their revenue but to justify it. There are also islands; you know; islands where you have to rent boats [canoe], which requires resources because they are often mobilized for entire days. We can rent a canoe for 2,000,000 GNF [$200 USD] per day…”. EIA 01, regional health system manager, male. A third best practice was advocacy with local authorities for the mobilization of additional material and logistical resources. It was explicitly reported that local authorities were involved in mobilizing means of transportation (motorcycles and canoes) to facilitate the access of distribution agents to remote locations

“For example, if we say that a team must reach 50 people, you will find that there is a village 20-25 km away and there are only 5 people there, so you need a special motivation. The islands too, there are difficult to access. Perhaps the programme says that we take only 5 islands whereas there are 15 islands in Boké. It is necessary then to sensitize those who have the canoes to help us. Or we can go to the local authorities to request support to reach these populations…”.

EIA 01, regional health system manager, male.

### 3.4. Obstacles Faced by Health System Personnel in Mass Distribution Campaigns for the Control of Lymphatic Filariasis

From the interviews with the participants, five main obstacles emerged that undermine the implementation of mass distribution campaigns for the control of lymphatic filariasis in the Boké health district.

The first obstacle was the centralized planning of the distribution campaigns, which could result in shortcomings or omissions in the estimation of resources needed for the campaign. However, health district and services managers undertook initiatives to reduce the effect of these omissions or shortcomings on the effective implementation of the campaigns. Among these adaptation measures were the reorganization of the supervision teams, the deployment of additional distribution agents, and the mobilization of additional resources (financial, human, and logistics) to support the campaigns.

“In the case of the islands, we find the means to send a boat to the islands or to rent a boat. To send a team more than 50 km away, we must send a motorcycle, if the service has a motorcycle. But if the service does not have a motorcycle, we have to rent it and pay for the fuel, and the person who goes there has to be given a bonus to encourage him.”

EIA 01, regional health system manager, male.

The second obstacle was the frequent stockouts of medicines in certain localities during the distribution campaigns. These drug stockouts were said to be maintained by uncontrolled movements of populations in the mining areas of the Boké health district. Faced with such a situation, the participants stressed that coordination and communication between teams of supervisors were often used to solve these problems of drug shortages.

“It is that there are movements of our population, often underestimated in mining areas, which leads to shortages of medicines, especially deworming medicines, and the quantity that comes is insufficient for the needs.”

EIA 04, health center chief, male.

The third obstacle was the reluctance of some communities to participate in the campaigns. This reluctance was reportedly due to the adverse events experienced by some children in Fria and Coyah following the administration of drugs (Praziquantel). Other participants also mentioned that this reluctance was related to false rumors about the effects of mass drugs and medical products administered during campaigns on the fertility of communities.

“People say that the medicine we give them affects women’s fertility…”.

EIA 11, community health worker, male.

The fourth obstacle was the difficulty of accessing certain areas of the Boké health district, particularly the remote areas and the islands. Regarding the difficulty of access to certain localities, we provide the following quote from a community health worker:

“The sectors are too far apart. However, if it is not for the community, we cannot accept that. If you have to go to a locality where you are going to pay 40,000 GNF [$4USD] for transport to and from the location, if you are given 50,000fg [$5USD] what are you going to do with that? So, responsible of the health district need to take into account the transportation of the people who travel according to the distances they cover.”

EIA 15, community health worker, female.

Finally, it emerged that the implementation of mass distribution campaigns for the control of lymphatic filariasis often coincided with periods of intense agricultural activity by the population.

“If it is at the time of the rainy season, at the time of crops growing when we go to certain villages, it is difficult to find people”

EIA 03, community health worker, male.

**Figure 2:**
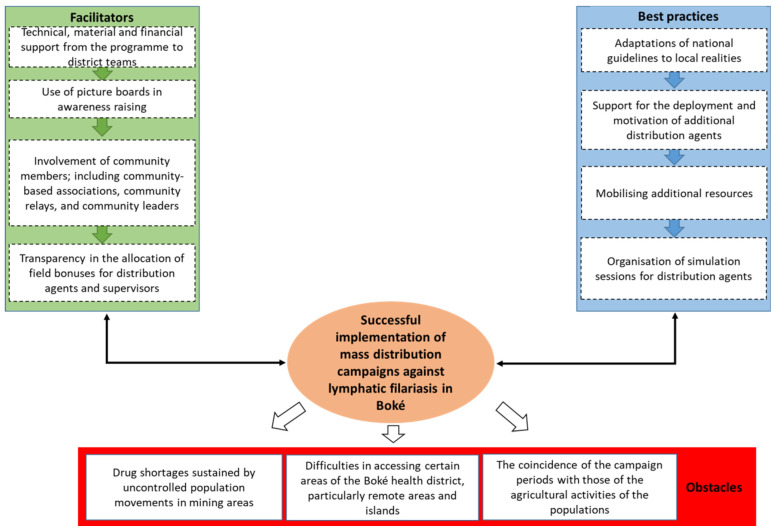
Summary of facilitators, best practices, and barriers to implementing mass distribution campaigns for lymphatic filariasis control in the health district of Boké, Guinea, in 2022.

The facilitators, best practices, and obstacles are summarized in Figure 2.

## 4. Discussion

This study explored the facilitators, best practices, and barriers to the implementation of mass distribution campaigns for the control of lymphatic filariasis in the health district of Boké, Guinea. Our results show that the involvement of community members in drug administration and the adaptation of national guidelines to local realities were respectively key facilitators and best practices for the success of the distribution campaigns in Boké. However, drug shortages during the campaigns were noted as one of the main obstacles to the effective implementation of distribution campaigns in Boké.

This study has one main limitation that should be noted. It is possible that our data are associated with (recall) bias since some of the distributors surveyed had not been involved in drug distribution for 2 years.

The involvement of community members, particularly community health workers, was an important facilitator of successful mass distribution campaigns for the control of lymphatic filariasis in Boké. In the Guinean context, community outreach workers are recruited by the communities themselves under the supervision of local health service managers (heads of health centers). Their selection follows rigorous criteria such as being able to read and write, having a good reputation within their community, being motivated and able to serve their community, and being able to speak the local language. The use of community members in the administration of drugs in their communities contributed to the fight against community reluctance to participate and adhere to medication and false rumors. The use of community members in drug distribution has already been documented in Kenya as an effective means of communication and community adherence to lymphatic filariasis control campaigns [2]. Silumbwe et al. in their systematic review also highlighted that raising awareness through innovative community participation programmes and building partnerships and collaborations with community members in organizing campaigns were facilitators in the implementation of mass distribution campaigns against filariasis in sub-Saharan Africa [2]. The findings of our study emphasize the need for decision-makers in the national programme for NTD control to safeguard the involvement of community members in mass distribution campaigns. National policies and health system actions should also work to improve the motivation of community health workers to achieve the national objectives of the fight against NTDs. The motivation needed could include the provision of equipment (torch, bag, telephone, etc.) and means of transportation (bicycle, motorcycle, etc.). This recommendation is all the more obvious as findings from this study show how difficult working conditions may be, for example, long distances from the villages and blocks to be covered, poor road networks, insufficient incentives, etc.

Our study also shows that the adaptation of national guidelines for mass distribution campaigns to local realities was an essential best practice for the effective implementation of lymphatic filariasis distribution campaigns in Boké. The adaptation of national guidelines included the redefinition of the areas to be covered, the composition of supervisory teams, and the local mobilization of additional resources (logistical, human, financial, etc.) to achieve campaign coverage objectives. The findings reflect the experience, positive attitude, and motivation of the managers of the health system and services in Boké for the implementation of mass distribution campaigns.

The findings also call on the key personnel of the national programme for NTD control and their partners to work towards decentralizing the planning and implementation of distribution campaign activities. Indeed, the readjustments of directives to overcome the shortcomings in the central planning of campaign activities played an important part in the overall success of the activity. For example, faced with insufficient human and financial resources to cover all areas of the Boké health district, an area endemic for filariasis, the implementing health system personnel resorted to using revenues from the health centers. In the short and medium term (1–3 years), joint planning of campaign activities with local health system personnel should be considered by national officers for the control of NTDs. In the long term, decentralization of the planning and execution of campaign activities to the district level should be considered by the national NTD programme. In such an organizational arrangement, the national NTD control programme would have the role of mobilizing resources, supervising campaigns, and evaluating the impact of these campaigns, among other things. All of this would contribute to anticipating potential undesired effects (e.g., weak financial and organizational autonomy of health centers) on local health structures and to sustaining the achievements of the national programme for the control of NTDs in order to reach national control objectives. These recommendations on the progressive decentralization of NTD control activities are relevant to consider as mass distribution campaigns are increasingly seen as non-essential to interrupting lymphatic filariasis transmission in West Africa due to low levels of disease transmission in the region [5].

Frequent drug shortages during distribution campaigns were noted as a major obstacle to the effective implementation of mass distribution campaigns in Boké. This obstacle has already been documented by other authors in Africa [13,14]. In our study, this result was related to uncontrolled population movements in the mining areas of Boké, leading to miscalculations in population estimates. This finding of frequent drug shortages suggests the need for the national NTD programme to provide buffer stocks for Boké district, and other similar areas in Guinea, during mass distribution campaigns.

## 5. Conclusions

The results of this study show that there are several factors that determine the success of mass distribution campaigns for the control of lymphatic filariasis in the Boké health district. These factors include, among others, the involvement of communities in the distribution of drugs, the significant mobilization of human, financial, and logistic resources, and the existence of experienced and motivated local teams for the execution of campaign activities. However, the frequent shortages of medicines, the difficulty of access to rural areas, and the lack of logistical means for the supervision of activities are the main obstacles to the success of the various mass distribution campaigns in Boké. The provision of buffer stocks for special areas such as Boké by national programme officers and partners, joint planning of campaign activities with local managers of health systems and services, and improvement of existing mechanisms for motivating health workers, including community health workers, during future campaigns should help to achieve national objectives in the fight against NTDs in Guinea.

## Figures and Tables

**Table 1:** Profile of health system personnel participating in the study, Boké health district, N = 18.

Profile	Number (%)
Health system and services managers	03 (17)
Skilled healthcare providers	10 (55)
Communities (community leaders and community health workers)	05 (28)
Sex	
Men	14
Women	4

## Data Availability

The data presented in this study are available on request from the corresponding author. The data are not publicly available due to confidentiality and security issues.
